# Cryptic *in vitro* ubiquitin ligase activity of HDMX towards p53 is probably regulated by an induced fit mechanism

**DOI:** 10.1042/BSR20220186

**Published:** 2022-07-04

**Authors:** Karla Gisel Calderon-González, Ixaura Medina-Medina, Lucia Haronikova, Lenka Hernychova, Ondrej Bonczek, Lukas Uhrik, Vaclav Hrabal, Borivoj Vojtesek, Robin Fahraeus, Jesús Hernández-Monge, Vanesa Olivares-Illana

**Affiliations:** 1 Laboratorio de Interacciones Biomoleculares y cáncer. Instituto de Física Universidad Autónoma de San Luis Potosí, Av. Parque Chapultepec 1570, Privadas del pedregal, 78210, SLP, México; 2 Regional Centre for Applied Molecular Oncology, Masaryk Memorial Cancer Institute, Zluty kopec 7, 656 53 Brno, Czech Republic; 3 Department of Medical Biosciences, Umeå University, SE-90185 Umeå, Sweden; 4 Équipe Labellisée Ligue Contre le Cancer, Université Paris 7, INSERM UMR 1162, 27 Rue Juliette Dodu, 75010 Paris, France; 5 Catedra CONACyT- Laboratorio de Biomarcadores Moleculares. Instituto de Física, Universidad Autónoma de San Luis Potosí, México City, México

**Keywords:** cancer, HDM2, HDMX, Induced fit, MDM2, MDMX, p53, ubiquitination

## Abstract

HDMX and its homologue HDM2 are two essential proteins for the cell; after genotoxic stress, both are phosphorylated near to their RING domain, specifically at serine 403 and 395, respectively. Once phosphorylated, both can bind the *p53* mRNA and enhance its translation; however, both recognize p53 protein and provoke its degradation under normal conditions. HDM2 has been well-recognized as an E3 ubiquitin ligase, whereas it has been reported that even with the high similarity between the RING domains of the two homologs, HDMX does not have the E3 ligase activity. Despite this, HDMX is needed for the proper p53 poly-ubiquitination. Phosphorylation at serine 395 changes the conformation of HDM2, helping to explain the switch in its activity, but no information on HDMX has been published. Here, we study the conformation of HDMX and its phospho-mimetic mutant S403D, investigate its E3 ligase activity and dissect its binding with p53. We show that phospho-mutation does not change the conformation of the protein, but HDMX is indeed an E3 ubiquitin ligase *in vitro*; however, *in vivo*, no activity was found. We speculated that HDMX is regulated by induced fit, being able to switch activity accordingly to the specific partner as p53 protein, *p53* mRNA or HDM2. Our results aim to contribute to the elucidation of the contribution of the HDMX to p53 regulation.

## Introduction

HDMX is a multi-domain protein; in its N-terminus contains a p53-binding domain, with a central acidic domain and a zinc finger; whereas, in the C-terminus there is a RING domain. It has been shown that HDMX does not present a nuclear localization signal (NLS) nor nuclear export signal (NES). However, under normal cellular conditions, the protein was found in the cytoplasm of cells, where together with HDM2 it promotes p53 polyubiquitination [1]. Under DNA damage conditions, HDMX is phosphorylated at serine 403 by the kinase ATM [2]. The form of HDMX phosphorylated at serine 403 (HDMXserine-403-P) was found at the p53 gene during its transcription, and it was observed that HDMXserine-403-P is able to bind the nascent *p53* mRNA in the nucleus of the cell, acting as an RNA chaperone in order to properly fold the *p53* mRNA for its further export by the HDM2 protein and translation [3]. HDM2, which is homologous to HDMX, is a multifunctional protein with a vast interaction network [4]. Indeed, HDM2 is recognized as a hub with more than 100 interacting partners among proteins and mRNAs [4]. HDM2 binds several mRNAs, such as p53 [5], XIAP [6], Slug [7], MYCN [8], RB [9] and E2F1 [10], and promotes their translations, acting as a translation factor with different cellular responses in each case. On the other hand, it can also bind a vast number of proteins, among them the products of the mRNAs that it binds and helps to translate, and tags them with the small ubiquitin-protein, since its C-terminal RING domain has E3 ubiquitin ligase activity. In particular, our group and others have reported that, under normal conditions, HDM2 binds p53 protein and acts alongside HDMX to poly-ubiquitinate p53 and stimulate its degradation [11]. Under DNA damage conditions, HDM2 is also phosphorylated by the kinase ATM, at serine 395 [12]. This post-translational modification (PTM) changes the protein conformation [13,14], resulting in the modification of its activity, since during genotoxic stress conditions HDM2 binds the *p53* mRNA and aids its translation [5,15,16].

Despite the high homology (over 72%) between the RING domain of HDMX and HDM2, HDMX has historically not been considered to be an E3 ubiquitin ligase [17–20]. Several groups have studied this apparent inconsistency; in 2010, Iyappan et al. published a study where through site-directed mutagenesis, they turn an HDMX without E3 ligase ubiquitin activity into a competent E3 towards p53. The substitution of two regions 448–453 aa by the respective region of HDM2 or a single mutant N448C and the region 465–480 aa, convert HDMX into a competent E3 for p53 [21]. The authors argue that these mutants allow a proper interaction with the UbcH5b E2 conjugating enzyme. In their study, they used recombinant GST-fused chimerical constructs. Recently, it has also reported a mice model expressing an inducible p53 allele HDM2 o HDMX deletions, the authors observed that HDMX but not HDM2 interacts with the UbcH5b E2 conjugating enzyme indicating the essential role of HDMX in p53 degradation in mice model [22]. It has been shown that the GST-tag can modify the properties of specific proteins [23]. In 2011, Wang et al. shown that the HDM2-GST construct was indeed able to poly-ubiquitinate p53, but in the absence of the tag, HDM2 performed the p53 mono-ubiquitination, whereas the presence of HDMX was indeed needed for proper p53 poly-ubiquitination [23,24]. In 2014, Ergorova et al. also performed site-directed mutagenesis to turn the HDMX RING domain into an active E3 ubiquitin ligase. In this case, they mutated N448C and K478R [25]. This study did not test the full-length HDMX protein, but a construct of the RING domain comprising residues 416–491. However, in 2002, Badciong and Hass showed that HDMX has an E3 ubiquitin ligase activity *in vitro*, and can autoubiquitinates itself and mediate the ubiquitination of p53 [26]. They also used an HDMX construct with GST-tag, which increases the discrepancies in the different results on the E3 ligase activity of HDMX.

In previous works, we expressed and purified the full-length of HDM2 and the phosphomimetic mutant HDM2 (S395D) with a small 6X His tag; through circular dichroism (CD) and *in vitro* ubiquitin ligase assays, we observed that the proteins were folded correctly, were active, and that the phosphomimetic mutation strongly alters the ternary structure of the protein [13]. Here, we express properly folded and active HDMX and HDMX(S403D) proteins that can ubiquitinated p53. However, no changes in the structure of the protein due to the phosphomimetic mutation were observed. We also compared the ability of the proteins and some constructs of the different domains to interact with p53 and to conserve the E3 activity.

## Materials and methods

### Plasmids and antibodies

Plasmids were prepared using the pET28a vector for bacterial expression and pcDNA vector for eukaryotic. The respective restriction sites were fused in each primer and used for each cloning. The cDNA for HDM2, p53, HDMX, HDMX(1-255), HDMX(322-490) and HDMXp60 were PCR amplified and cloned in the chosen vector. The mutagenesis of the S403D site was carried out by site-directed mutagenesis with the primer containing the mutated codon.

Proteins were resolved in 10% SDS-PAGE (sodium dodecyl sulfate-polyacrylamide gels) and then electrophoretically transferred to nitrocellulose membrane at 100 volts for 90 min at 4°C. The membrane was blocked with 5% non-fat dry milk in 1× PBS pH 7.4 for 1 h at RT. Then, the membranes were incubated with primary antibodies in 5% milk. For HDMX we employed anti-HDMX 255-11 (homemade antibody, 1:250) incubated for 1.30 h at RT. Later, the membranes were washed three times with PBS-T each 10 min at RT. Then, they were incubated with the secondary antibody anti-chicken (Millipore) at dilution 1:5000 for 1 h at RT, washed three times with PBS-T and revelated by chemiluminescence. The membranes were stripped with 1% sodium dithionite in 1× PBS overnight at 4°C, washed three times with PBS-T and blocked with 5% milk for 1 h a RT. Then, the membranes were incubated with primary antibody anti-p53 DO-1 (Santa Cruz) at dilution 1:1000 incubated for 2 h at RT, washed with PBS-T, incubated with secondary antibody anti-mouse 1:5000 for 1 h at RT and washed again with PBS-T. The signal of chemiluminescence was detected with a 1:1 dilution of ECL kit (Amersham) using autoradiography films (Carestream X-OMAT LS Film, Sigma-Aldrich). For the *in vitro* ubiquitination assay, we employed the primary antibody anti-ubiquitin (homemade antibody,1:500) in 7% of milk incubated 1 h at RT. The secondary antibody anti-chicken at dilution 1:5000 in 5% of milk, incubated 1 h a RT. In the case of p53, we used the antibody CM1(homemade antibody, previously reported [27]) at dilution 1:10,000 incubated 1 h a RT and the corresponding secondary antibody anti-rabbit 1:5000 in 3% milk.

### Expression of recombinant proteins

A pre-culture of 100 ml of 2XYT medium (bacto tryptone, yeast extract, NaCl pH 7.2) with kanamicyn was incubated overnight at 37°C in agitation. Then, cultures of 1000 ml of 2XYT medium with kanamycin were incubated at 37°C with agitation rate of 200 rpm until an optical density (OD600nm) between 0.9 and 1.0 was reached. The cultures were induced with 0.5 M of isopropil-β-D-1-tiogalactopiranoside (IPTG) as follows: HDM2, HDMX full-length, HDMX full-length S403D, HDMX C-ter 322, HDMX S403D C-ter 322, HDMXp60, HDMXp60 (S403D), HDMX (1-255) incubated at 37°C at 200 rpm for 3 h; HDMX-RING and p53 were incubated at 15°C at 200 rpm for 16 h. The cultures were centrifugated at 4300 rpm for 20 min a 4°C, the supernatant was discarded, and the pellets were kept a −80°C until use.

### Immunoprecipitation

Two mixed HDMX:p53 total lysate samples were incubated separately with anti-p53 (DO-1 mouse antibody, Santa Cruz Biotechnology) and anti-HMDX (D-4 mouse antibody, Santa Cruz Biotechnology) along with sepharose beads (rec-Protein G-Sepharose Conjugate, INVITROGEN) overnight at 4°C. The immunoprecipitants and total protein extracts were loaded onto SDS-PAGE and analyzed by Western blot for detection of p53 protein (CM1 rabbit antibody).

### Purification of recombinant proteins

HiTrap nickel columns (GE Healthcare, Chicago, IL, U.S.A.) were employed to protein purification. Briefly, the pellets were resuspended in buffer A (50 mM Tris, 200 mM NaCl, 40 mM imidazol, 10 μM ZnSO_4_, 10% glycerol and 10 mg of phenylmethanesulfonyl fluoride [PMSF], SIGMA) and sonicated 20 times in ice for 20 s at 40% amplitude in an Ultrasonic Processor (Cole Parmer), cooling for 20 s in ice between each sonication cycle. Subsequently, the samples were centrifugated at 10,000 rpm for 15 min at 4°C. After centrifugation, the supernatant was recollected (soluble fraction) and the pellet (insoluble fraction) was resuspended with 10 ml of urea buffer (urea 8 M, 50 mM Tris pH 8.5, 150 mM NaCl, 10 mM imidazole) and incubated 2 h a room temperature (RT) in gentle agitation. The soluble protein p53 was charged into the nickel column, washed with 75 ml of buffer A with 10 mM of β-mercaptoethanol and eluted with 5 ml of buffer B (50 mM Tris pH 8.5, 200 mM NaCl, 10 mM ZnSO_4_, 300 mM imidazole). In the case of the insoluble proteins (HDM2, HDMX full-length, HDMX full-length S403D, HDMX C-ter 322, HDMX S403D C-ter 322, HDMXp60, HDMXp60 [S403D], HDMX-RING and HDMX [1-255]), they were charged into the nickel column and wash β-mercaptoethanol and eluted with 5 ml of B buffer. Finally, the samples were concentrated by centrifugation in Amicon tubes (Merck Millipore) up to a volume of 100–200 μl. For the *in vitro* ubiquitination assay, a buffer exchange was done by adding 10 ml of phosphate buffer (10 mM H_2_NaPO_4_/ HNa_2_PO_4_ pH 8.0.) at the time of concentrating the samples with the centricons. The samples were quantified by Bradford assay and analyzed in SDS-PAGE. The proteins were visualized with Coomassie Blue stain.

### Enzyme-linked immunosorbent assay (ELISA)

Ninety-six well plates (Thermo scientific) were coated with 200 ng of p53 in 0.1 M sodium bicarbonate (NaHCO3) (50 μl/well) overnight at 4°C. After the incubation, the plates were washed six times with 200 μl of 0.1% (v/v) Tween 20 in 1× PBS (PBS-T), blocked with 3% albumin (BSA) (Sigma Aldrich) for 1 h at RT and washed four times with 200 μl of PBS-T. Subsequently, different dilutions from 0 to 1000 ng/μl of the HDMX binding protein (HDMX full-length, HDMX full-length S403D, HDMX C-ter 322, HDMX S403D C-ter 322, HDMXp60, HDMXp60 [S403D], HDMX-RING and HDMX [1-255]) were added into each well plate in 50 μl of 3% BSA and incubated 1 h at 4°C. The plates were washed six times with 200 μl PBS-T and incubated with primary antibody anti-HDMX (D-4) (Santa Cruz) at dilution 1:1000 for 1 h at RT. Later, the plates were washed again with 200 μl of PBS-T and incubated with the secondary antibody anti-mouse (homemade) at dilution 1:1000 for 1 h at RT. Finally, the plates were washed as above mentioned, incubated with 50 μl of ECL reagent (GE healthcare) in relatio 1:1 and the chemiluminescence signal was read with the plate reader Victor X4 (PerkinELmer).

### HDM2 knock-out in H1299 cells (H1299 ΔHDM2)

The H1299 ΔHDM2 cell line was constructed using the CRISPR Cas9 system; briefly, H1299 cells were co-transfected with the HDM2 CRISPR/Cas9 KO Plasmid (Cat # sc-sc-400045, Santa Cruz Biotechnology, Santa Cruz, CA, U.S.A.), which encodes for the RNA guide and the RNA guided-nuclease specific for human species, as well as with the HDM2 HDR Plasmid (h2) (Cat# sc-400045-HDR-2, Santa Cruz Biotechnology, Santa Cruz, CA, U.S.A.) that encodes for a homology-directed DNA repair template corresponding to the cut sites generated by the HDM2 CRISPR/Cas9 KO plasmid. The HDM2 HDR plasmid also incorporates a puromycin resistance gene that allow the selection of stable knockout cells. The single-cell clones were obtained by serial dilution and were selected with 3 μg/ml of puromycin. The clones were tested for HDM2 expression through Western blot analysis and only those negatives for HDM2 protein expression were expanded.

### *In vitro* ubiquitination assay

Ubiquitination reactions contained 25 mM of HEPES pH 8.0, 10 mM of MgCl_2_, 4 mM of ATP, 0.5 mM of DTT, 0.05% (v/v) of Triton X-100, 0.25 mM of benzamidine, 10 mM of creatine phosphate, 3.5 units/ml of creatine kinase, ubiquitin or His-tagged ubiquitin (2 μg) and E1 (100 nM), E2 (1 μM) (UBPBio, Dallas TX, U.S.A.) and the proteins of interest; p53 at 5 μg, HDMX full-length, HDMX full-length S403D, HDMX C-ter 322, HDMX S403D C-ter 322, HDMXp60, HDMXp60 (S403D), HDMX-RING and HDMX (1-255) at 10 μg. Reactions were started with the addition of HDM2 or HDM2 (241-491), incubated for 15 min at 30°C, and analysed with 10 and 15% SDS–PAGE followed by immunoblotting.

### CD spectroscopy

The circular dichroism (CD) spectra of HDMX and HDMX (S403D) were recorded using a Jasco J-810 spectropolarimeter in a wavelength range of 200–280 nm for far-UV CD and 250–310 nm for near-UV CD. The protein concentration was 0.5 mg/ml in phosphate buffer, pH 8, and quartz cuvettes of 0.1 cm path length were used.

### Intrinsic fluorescence

The emission fluorescence spectra of the proteins at 0.5 mg/ml in phosphate buffer, pH 8, were recorded in a Perkin-Elmer LS-55 spectrofluorometer in quartz cuvettes of 1 cm path length at room temperature. The excitation wavelengths were 280 and 295 nm. Blanks without protein were recorded and subtracted from the experimental spectra.

### Hydrogen/deuterium exchange (HDX) mass spectrometry

#### Sample preparation

For each experiment, proteins were diluted with buffer (50 mM Tris-HCl [pH 7.5]; 150 mM NaCl, 2 mM DTT) in H_2_O to prepare undeuterated controls and for peptide mapping. The deuterated samples were preincubated for 20 min on ice and then diluted with the buffer in D_2_O (50 mM Tris-HCl (pH 7.1); 150 mM NaCl, 2 mM DTT). HDMX wt and HDMX-S403D were measured at final concentrations 1 µM, hydrogen-deuterium exchange was carried at room temperature and quenched after 10, 60 and 1800 s by quickly adding quench buffer (0.5 M TCEP-HCl), 1 M glycine (pH 2.3) and pepsin (1 mg/ml) at 10:3:5 (TCEP-HCl:glycine:pepsin, v/v/v) ratio followed with 3 min incubation and rapid freezing in liquid nitrogen. For the measurement of HDMX–p53 interaction, HDMX samples at final concentrations 1 µM alone and with p53 in a molar ratio 1:3 (HDMX:p53) were prepared, and hydrogen-deuterium exchange was carried at room temperature and quenched after 60 s as described earlier.

#### Sample measurement

Each sample was thawed and injected into an LC system (UltiMate 3000 RSLCnano, Thermo Scientific) with immobilized Nepenthesin-1 and pepsin enzymatic columns (Affipro s.r.o., CZ) 15 µl bed volume flow rate 100 µl/min, 2% acetonitrile/0.05% trifluoroacetic acid. Peptides were trapped and desalted on-line on a peptide microtrap (Michrom Bioresources, Auburn, CA, U.S.A.) for 3 min at a flow rate of 100 µl/min. Next, the peptides were eluted onto an analytical column (Jupiter C18, 0.5 × 50 mm, 5 µm, 300 Å, Phenomenex, CA, U.S.A.) and separated by linear gradient elution starting with 10% buffer B in buffer A and rising to 40% buffer B over 28 min at a flow rate of 50 µl/min. Buffers A and B consisted of 0.1% formic acid in water and 80% acetonitrile/0.08% formic acid, respectively. The immobilized Nepenthesin-1 and pepsin columns, trap cartridge, and analytical column were kept at 1°C.

#### Data analysis

Data analysis was carried out as previously described (citation: PMID: 31652301). Data were graphically visualized with the MS Tools application (citation: http://dx.doi.org/10.1016/j.ijms.2010.07.030).

## Results

### Phosphorylation on serine 403 of HDMX does not change its structure

**Figure 1:**
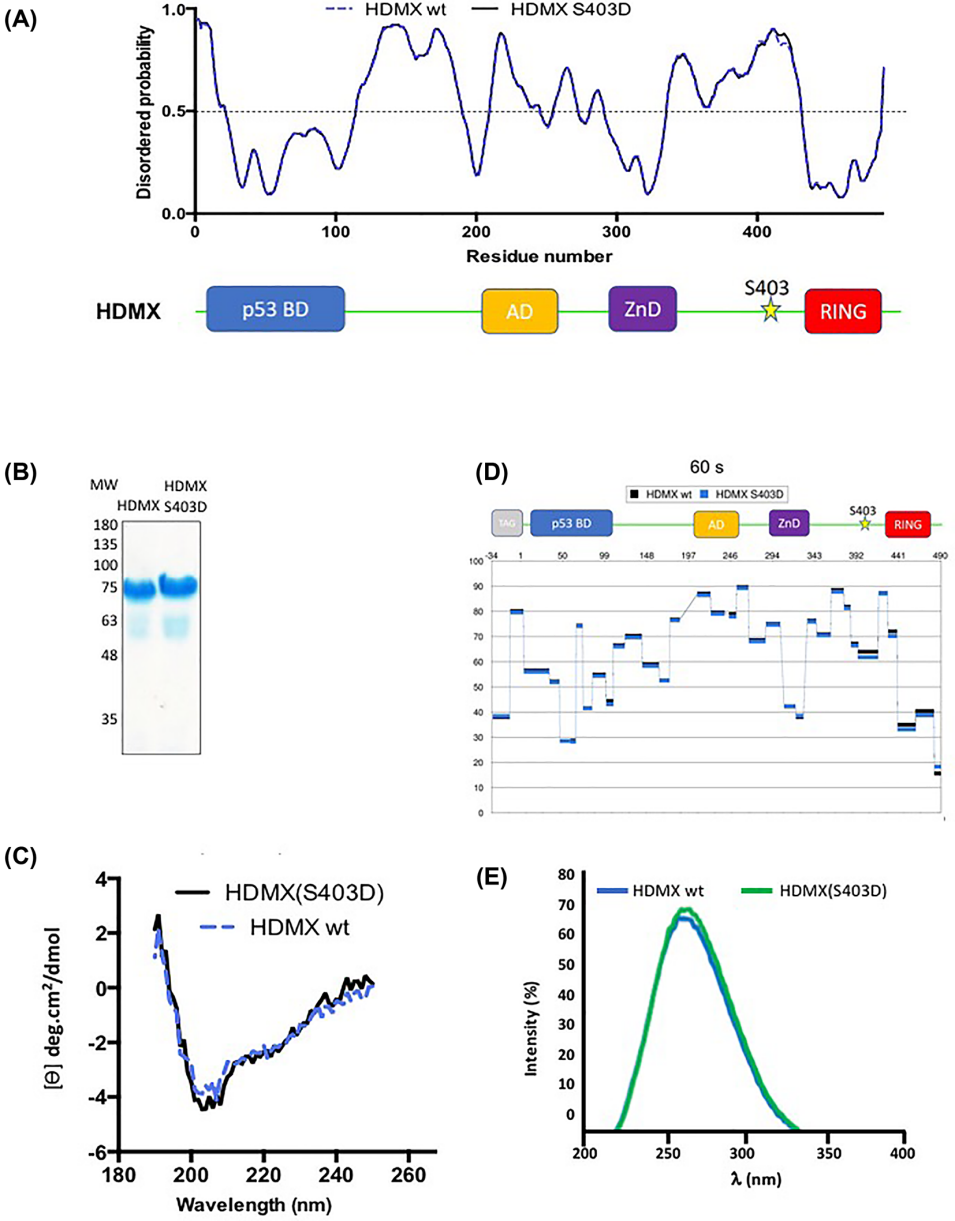
HDMX does not show conformational rearrangement by phosphomimetic mutant HDMX(S403D) (**A**) The predicted intrinsically disordered regions of HDMX (dotted blue line) and HDMX (S403D) (black line) are shown in the upper panel [28]. Cartoon illustrating major domains of HDMX; p53 BD, the p53 binding domain (blue); AD, acidic domain (yellow); ZnD, zinc finger domain (violet); RING, really interesting new gene domain (red) the star represents the place of the phosphomimetic mutation S403D. (**B**) Recombinant purified HDMX and HDMX(S403D). MW, molecular weights in kDa. (**C**) Far-UV CD spectra of the HDMX and phosphomimetic mutant HDMX(S403D). (**D**) HDMX and HDMX (S403D) mutant difference in deuteration, the data are plotted as % of deuteration of the peptide as a function of the numbering of the amino acids -34–490 after 60 s of incubation in the deuterated buffer. The -34 to 1 is due to the 6XHIS tag. For other incubation times, see Supplementary Figure S1. (**E**) The intrinsic fluorescence emission spectra of HDMX and phosphomimetic mutant HDMX(S403D). The spectra shown were obtained after subtracting the blank (no enzyme) from the experimental values. One representative experiment of three is shown.

HDMX is a 490 aa long protein that contains several folded domains connected by intrinsically disordered regions (IDR) (Figure 1A) [28]. Several domains were solved by X-ray crystallography or NMR (PMID: 18219319, PMID: 26148237, PMID: 26812210, PMID: 19153082), but the structure of the whole protein is still missing. The ATM-dependent phosphorylation of serine 403 falls in the last IDR between the Zn finger and the RING domain, similar to the phosphorylation of HDM2 at serine 395. Under normal conditions, HDMX helps HDM2 to properly poly-ubiquitinate p53 to stimulate its degradation; however, after genotoxic stress, the kinase ATM phosphorylates HDMX, switching its activity towards acting as a *p53* mRNA chaperone [3]. It has been shown that the phosphomimetic mutation S395D dramatically changes the protein’s conformation [13,14], which explains the switch from ubiquitin ligase towards translation factor for p53. We decided to investigate whether the ATM-mediated phosphorylation of HDMX at S403 also changes the protein conformation, similar to what happens with HDM2. The phosphomimetic mutation HDMX (S403D) has been shown to mimic the phosphorylation of the protein [3]. We then produced the HDMX wild-type (wt) and the phosphomimetic mutant in an *Escherichia coli* recombinant system and purified them (Figure 1B) in order to characterize them structurally. In the results of Far-UV circular dichroism (CD), no change in the two proteins' secondary structures was observed (Figure 1C). A deuterium/hydrogen exchange mass spectrometry (HDX-MS) experiment was conducted, exhibiting no differences between the wild-type and the S403D mutant at 60 s deuteration interval (Figure 1D) or in other deuteration intervals (10 s and 1800 s, Supplementary Figure S1). The intrinsic fluorescence analysis of the proteins was performed to corroborate the previous results and shows no changes in ternary conformation either (Figure 1E). Taken together, the results show that the phosphomimetic mutation does not alter the conformation of the protein either in its secondary structure or the global conformation of the whole protein; these results were very surprising since they contrast with the previously published results on its homologue HDM2 [13,29].

### The HDMX plasticity and the effect of the phosphomimetic mutation on HDMX–p53 interaction

**Figure 2:**
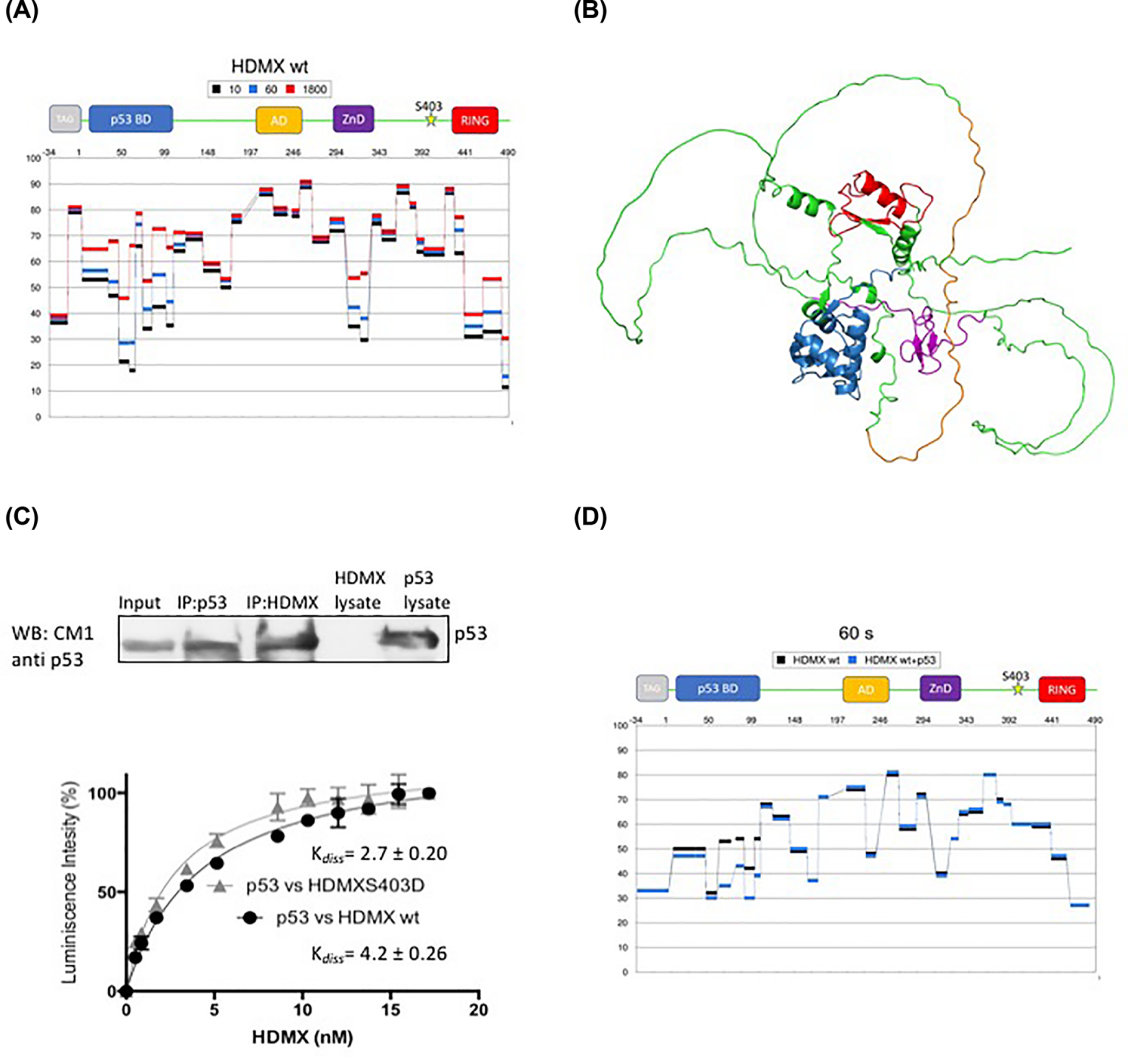
HDMX is a very flexible protein (**A**) HDX-MS of HDMX at different deuteration times, the data are plotted as % of deuteration of the peptide as a function of the numbering of the amino acids -34–490 after 10 s (in black), 60 s (in blue) and 1800 s (in red) of incubation in the deuterated buffer. The data shown represent the averages and standard deviations (SD) from five independent experiments. (**B**) Molecular modeling of HDMX using the AlphaFold Protein Structure Database (PMID: 34265844, PMID: 34791371) [30,31]; the p53 BD is shown in blue, the zinc finger in violet and RING in red are structurally ordered whereas the acidic domain in orange is part of the intrinsically disordered, other IRG are shown in green. (**C**) In the upper panel; recombinant p53 and HDMX proteins were synthesized from *E. coli* by separate. Then, a mix of equivalent protein quantities was used for immunoprecipitation with anti-p53 and anti-HDMX. Note that anti-p53 does not react with HDMX lysate. Lower panel; ELISA using a fixed amount of recombinant purified p53 (10 ng/μl) and increasing amounts of HDMX or HDMX(S403D) (0–20 ng/μl). HDMX and HDMX(S403D) show similar affinity towards p53, with *K*_d__iss_ values of 4.4 and 2.7, respectively. (**D**) HDX MS of HDMX (in black) and HDMX-p53 interaction (in blue). The data are plotted as % of deuteration of the peptide as a function of the numbering of the amino acids (-34 to 1 is due to the 6XHis Tag) 1–490 after 60 s of incubation in the deuterated buffer.

HDX-MS is a valuable method for protein structural changes monitored under physiological conditions in solution. We used HDX in three-time intervals to unveil the nature of different HDMX domains (Figure 2A). The gradual incorporation of deuterium to the amide backbone is seen for p53 binding domain (aa 19–102), Zinc finger domain (aa 290–332) and RING domain (aa 437–483) indicating that these parts are structurally ordered and dynamic. Interestingly, the acidic domain (aa 215–255) behaves more like disordered regions of the protein (in concordance with the seen in Figure 1A), where the deuteration is high and do not change in time as these protein parts are exposed more to the solvent. The AlphaFold Protein Structure Database (PMID: 34265844, PMID: 34791371) was used to model the whole protein structure and confirmed the HDX results (Figure 2B) [30,31]. The p53 BD (blue), zinc finger (violet) and ring (red) are structurally ordered whereas the acidic domain (orange) remains in unfolded state. Altogether, these results predict HDMX protein to be very flexible and dynamic interacting molecule.

It has been reported that HDMX binds p53 through different domains. It was first shown that, like HDM2, HDMX harbours a p53-binding domain in the N-terminal part of the protein (Figure 1A) and is able to interact with the N-terminal domain of p53 [32–34]. Later, it was shown that after HDMX phosphorylation at serine 289, the acidic and RING domains are also able to interact with p53 [35]. Then, we assayed the interaction between p53 and HDMX and the phosphomimetic mutant (S403D) in order to analyse whether the serine 403 phosphorylation has an inhibitory or enhanced role in the interaction. First of all, we used a co-immunoprecipitation assay to evaluate the interaction between HDMX and p53. Producing the proteins in *E. coli*, the extract of each one was mixed and immunoprecipitated for p53 or HDMX, and we observed the interaction between p53 and HDMX (Figure 2C, upper panel). Using an ELISA assay, we evaluated the effect of the phosphomimetic mutation (S403D) on the p53 interaction. We coat a plate with a fixed amount of p53 and add increasing concentrations of HDMX wt or the phosphomimetic (S403D) protein. The interaction does not present any significant differences in the binding of the two versions of HDMX to p53 (Figure 2C, lower panel). These results are concordant with the previous experiment that shows that the phosphomimetic mutation does not affect the global structure of HDMX. This also show that HDMX itself is flexible and its structure depends on the interacting partner supporting the idea of this induced fit interaction. Next, using HDX-MS, we evaluated the change in deuterium-hydrogen exchange in HDMX during its interaction with p53 at the 60 s, and as we observed that the main differences between HDMX alone and HDMX–p53 are found in the N-terminal part of the HDMX protein, which fits perfectly with the well-known p53 box 1-HDMX N-terminal interaction (Figure 2D). We expected to see some secondary interactions, it is probably that these secondary interfaces are more dynamic or transient and our HDX method isn´t able to capture them, this fit with the idea that HDMX is a very flexible protein.

### Dissecting the p53–HDMX interaction

**Figure 3:**
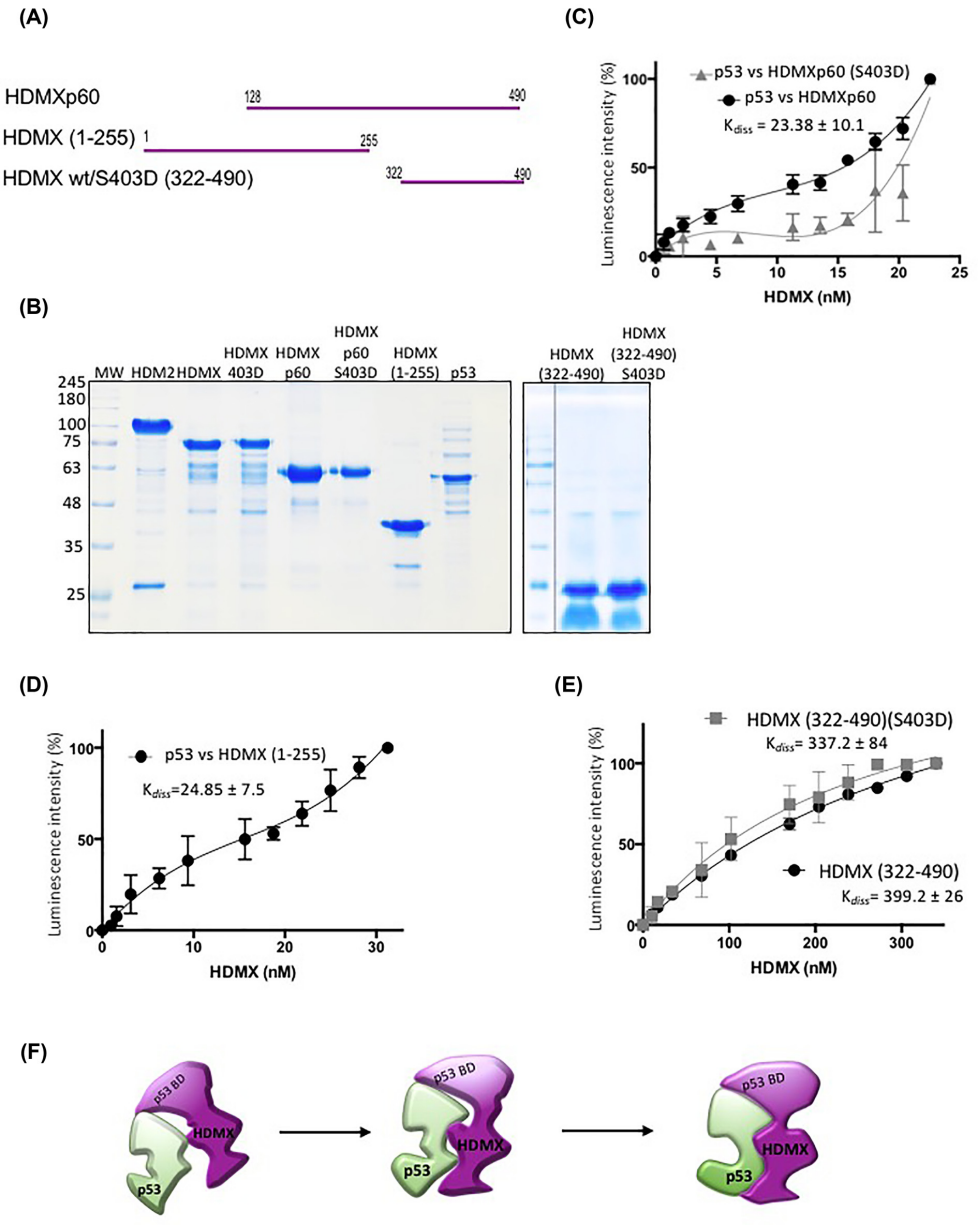
The different domains of HDMX bind p53 protein (**A**) The different construct using to dissect the interaction with p53. (**B**) SDS-PAGE of the recombinant purified constructs of HDMX, p53 and HDM2; MW, molecular weights in kilodaltons. (**C**) ELISA using a fixed amount of recombinant purified p53 (10 ng/μl) and increasing amounts of HDMXp60 or HDMXp60(S403D) (0–20 ng/μl). (**D**) ELISA using a fixed amount of recombinant purified p53 (10 ng/μl) and increasing amounts of HDMX(1-255). (**E**) ELISA using a fixed amount of recombinant purified p53 (10 ng/μl) and increasing amounts of HDMX (322–490) or HDMX (322–490)(S403D) (0–20 ng/μl). (**F**) Model of interaction between HDMX and p53 showing the flexibility of HDMX and the induced fix interaction.

In order to observed the secondary interactions between p53 and other domains different from the p53 binding domain we prepared a series of HDMX constructs to test their p53 binding activity. The first is an N-terminal-lacking protein; it starts at residue 128 to 490 and has been reported as a natural HDMX isoform derived from a cap-independent translation N-terminal truncated form named HDMXp60. This isoform initiates at the 7^th^ AUG codon downstream of the first AUG of the full-length HDMX [36]. This construct lacks the canonical p53 binding activity, but conserves the serine 403. Then, we prepared the wild-type and the phosphomimetic mutant (S403D) of this isoform. The second constructs start at the first residue but finish at residue 255, lacking the zinc finger and RING domains responsible for the E3 ubiquitin ligase activity, named HDMX(1–255). The third and fourth are small constructs of the C-terminal part of the protein. Both start at reside 322 and continue to the end, but one is the wild-type, and the other is the S403D mutant; they are named HDMX(322–490)-wt/S403D (Figure 3A).

We expressed and purified all constructs (Figure 3B) and used them in ELISA assays to test their ability to interact with p53 protein. The ELISA plates were coated with recombinant p53, and increasing concentrations of each protein were added. To our surprise, all of them bind the full-length p53 protein, however, with different affinities. HDMXp60, despite being able to interact with p53 shows a sigmoidal behaviour with a *K*_diss_ of 23.3 nM. The phosphomimetic mutant binds poorly at low HDMXp60 (S403D) concentrations; and high concentrations of the protein are required in order to observe a good interaction. The phosphomimetic mutation has an effect on this protein, indicating that the lack of the N-terminal part of the protein affects the C-terminal RING in an allosteric manner (Figure 3C). The construct HDMX(1–255) lacks the RING domain and Zn finger, and as expected, since it is conserved, the N-terminal domain binds very well to p53, with a *K*_diss_ of 24.85 nM, very similar to HDMXp60 (Figure 3D). Next, we tested the construct of the C-terminal part of the protein that preserves the phosphorylation site and the RING domain. In both cases, we observed interaction with the p53 protein (Figure 3E); the HDMX(322–490)-wt and the phosphomimetic construct HDMX(322–490)-(S403D) bind with the same affinity, with *K*_diss_ of 399 and 337 nM, respectively. This result contrasts with that observed for HDMXp60. A possible explanation of these differences is that HDMX (322–490) lacks the acidic domain and the Zinc finger, which can regulate the overall structure of the protein. Taken together, these results suggest that the first recognition site is the N-terminal part of the protein; however, this is not the only one. The N-terminal part has allosteric behaviour along with the C-terminal part of the protein, as we could see with HDMXp60, but the acidic domain and the zinc finger also regulate the binding, and the whole structure of the protein participates in the interaction with p53 showing an induced fit interaction with p53 (Figure 3F).

### HDMX E3 ubiquitin ligase activity towards p53

**Figure 4:**
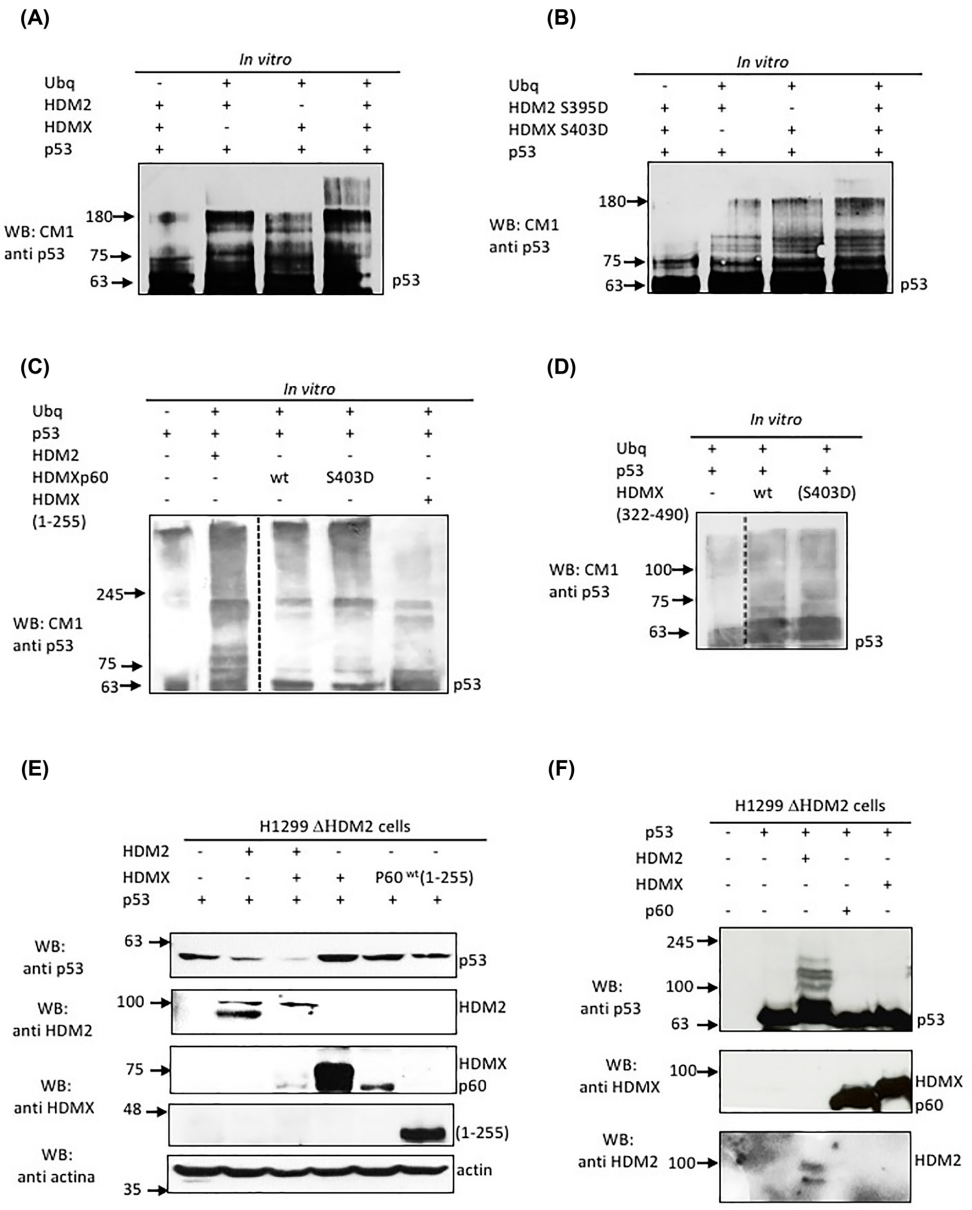
Cryptic HDMX ubiquitin ligase activity towards p53 (**A**) *In vitro* ubiquitination of recombinant p53 with HDM2 and/or HDMX. (**B**) *In vitro* ubiquitination of recombinant p53 with HDM2(S395D) and/or HDMX(S403D). (**C**) *In vitro* ubiquitination of recombinant p53 with HDM2 or HDMXp60 and the phosphomimetic mutant, and HDMX (1-255). (**D**) *In vitro* ubiquitination of recombinant p53 with HDMX (322-490) wt and the phosphomimetic mutant. (**E**) Levels of expression of p53 in H1299 ∆HDM2 cell line transfected with the HDM2, HDMX, HDMXp60 and HDMX (1-255). (**F**) *In vivo* ubiquitination in H1299 ∆HDM2 cell line of p53 with HDM2, HDMX or HDMXp60 using MG 132 to inhibits proteasome activity.

After showing that our recombinant HDMX protein was properly folded and able to bind p53, we decided to analyse whether the protein contained an E3 ubiquitin ligase in its RING domain. We used recombinant UbcH5c E2 conjugating enzyme, ubiquitin, HDM2 and HDMX, and together with the proper buffer, the reaction was assembled and incubated for 30 min at 37°C. In Figure 4A, we can observe the *in vitro* ubiquitination of p53 (Figure 4A, lane 1, p53 control). The p53 HDM2-dependent ubiquitination profile can be observed (Figure 4A, lane 2). The HDMX-dependent ubiquitination profile of p53 is shown (Figure 4A, lane 3). In the last lane, the HDMX-HDM2 dependent p53 ubiquitination is shown (Figure 4A, lane 4). We can observe that the HDMX recombinant protein is indeed able to ubiquitinate p53; another important observation is that, as has been reported, HDM2 and HDMX act together to properly poly-ubiquitinate p53. Next, we prepared the phosphomimetic mutants HDM2(S395D) and HDMX(S403D) to assay them in an *in vitro* ubiquitination assay towards p53. As we can see in Figure 4B, the mutants of HDMX also have E3-ubiquitin ligase activity; HDM2(S395D), as we reported previously, is able to ubiquitinate p53 in the absence of the *p53* mRNA [13] (Figure 4B). It is important to note that the higher molecular weight forms of p53 poly-ubiquitination carried out by the hetero-oligomer HDMX-HDM2 is no longer observed with the phosphomimetic form of the proteins. However, as we observed in Figure 1, no significant differences were observed in the structure between the HDMX-wt and the S403D. Thus, one possible explanation of this phenotype is that the HDM2 protein undergoes a conformational change under stress conditions and with the phosphomimetic mutant (S395D), and it is HDM2 that drives the structure of the hetero-oligomer, therefore changing the profile of p53 ubiquitination.

Furthermore, we tested the ability of HDMXp60-wt and HDMXp60 (S403D), the C-terminal constructs (322–490) wt/(S403D) and HDMX(1–255) to ubiquitinate p53. Then in Figure 4C, we show the p53 profile of ubiquitination HDM2-dependent. The HDMXp60 and HDMXp60 (S403D), both, present ubiquitination activity towards p53. HDMX (1–255), which strongly binds to p53, is not able to ubiquitinate p53. This is expected because it has no RING domain activity (Figure 4C). The C-terminal constructs HDMX (322–490) wt and (S403D) are both able to ubiquitinated p53 with the same profile (Figure 4D and Supplementary Figure S2).

Finally, we test the ability of HDMX to ubiquitinate p53 in cells. We used the H1299 cell line previously treated with CRISPR/cas9 to knock-out HDM2 (H1299 ∆HDM2). Then, we transfected this cell line with the p53 and some of the following constructs: HDMX, HDM2, HDMXp60 or HDMX(1-255); after 48 h post-transfection, we collected the cells and observed the p53 levels. As we expected, the construct HDMX (1–255) does not change the p53 levels compared with the control. However, neither HDMXp60 nor HDMX show any changes. The p53 levels decrease with HDMX only in the presence of HDM2. We observed a p53 decrease with HDM2 alone (Figure 4E). To corroborate this result, we transfected the cells or not with p53, HDM2, HDMX and HDMXp60, and treated them with the MG132 in order to abolish the proteasomal activity and accumulate the ubiquitinated form of p53. When we transfected with p53 and HDM2, we observed the p53 ubiquitination profile in lane 3. However, when we transfected with p53 and HDMXp60 or HDMX, no ubiquitination was observed at all. It is important to note that there is no detectable HDMX expression in the cell line that has not been transfected with HDMX (Figure 4F).

## Discussion

HDMX and its homologue HDM2 are both essential proteins during embryonic development, since mouse models lacking HDM2 or HDMX die in the early stages of development [37], the embryonic lethality due to the lack of HDM2 is rescued by the loss of p53 [38,39], deletion of HDMX also promotes embryonic lethality rescued by the loss of p53 [40]. HDMX and HDM2 proteins form a heterocomplex that, under normal conditions, poly-ubiquitinates p53. However, after genotoxic stress, both are a target for the ATM kinase protein and are phosphorylated at serine 403 and 395, respectively. The PTM switches its activity and together binds the *p53* mRNA and promotes its translation. Even though these activities have been clearly demonstrated, the details of the mechanism that regulates HDMX–HDM2–p53 under different conditions are not really well understood. In this work, we try to shed light on the p53 regulation by HDMX and HDM2.

The results show that HDMX, contrary to its homologous protein HDM2, does not change its conformation due to the phosphomimetic mutation S403D. The recombinant HDMXwt and HDMX (S403D) proteins are able to bind p53 and all the constructs tested are also able to bind the tumour suppressor protein. Each construct lacks a specific domain; HDMX (1–255) keeps the p53 binding domain, and can therefore recognize the protein. However, HDMXp60 and the C-terminal constructs that lack this important domain are all able to bind p53. In particular HDMXp60 and HDMX (1–255) have similar *K*_diss_ of 23.38 and 24.8 nM, respectively. Our results show that the C-terminal HDMX (322–490), the wild-type or the phosphomimetic mutant are still able to form a heterodimer with p53 but with a higher *K*_diss_ of 399 and 337, respectively. These results are in line with a previous study in which it was revealed that HDMX, HDM2 and p53 form a ternary complex involving the C-terminal domain, and even in the absence of the p53-binding domain, the trimer still forms and is able to polyubiquitinate p53 [11].

On the other hand, the natural HDMXp60 isoform was the only construct that presented a slight difference between the wild-type and the phosphomimetic mutant concerning p53 recognition, and further research in this subject is required. Finally, we also show that the recombinant HDMX presents E3 ubiquitin ligase activity *in vitro* together with all constructs that keep the C-terminal RING domain. The heterodimer HDMX/HDM2 wild type shows a different p53 profile of ubiquitination with a higher molecular weight than the heterodimer S403D/S395D. However, E3 activity can no longer be detected in H1299 ∆HDM2 cells transfected with the different constructs.

The lack of conformational change due to the phosphomimetic mutant HDMX (S403D) was astonishing since HDM2 (S395D) suffered a considerable change in conformation compared with the wild-type protein. This, together with the lack of HDMX E3 ubiquitin ligase activity *in vivo* conditions, suggested that HDMX is adapting factor to its partner protein and that the ubiquitination activity is HDM2 which drives the function. This is evidence of the importance of the heterodimer HDMX-HDM2.

Taken together, the results suggest that HDMX is a very plastic ‘helping factor’ that adapts itself to other proteins such as p53 or HDM2, and the binding with its partners produces an induced fit to accomplish a specific function. However, many questions are still open in the understanding of the role of HDMX in cells.

## Supplementary Material

Supplementary Figures S1-S2

## Data Availability

No primary data sets have been generated or deposited.

## Abbreviations

CD: circular dichroism
ELISA: enzyme-linked immunosorbent assay
HDX: hydrogen/deuterium exchange
IPTG: isopropil-β-D-1-tiogalactopiranoside
PTM: post-translational modification
