# Supplementary material for: Cryptic in vitro ubiquitin ligase activity of HDMX towards p53 is probably regulated by an induced fit mechanism
Source: Biosci Rep. 2022 Jul 4;42(7):BSR20220186. doi: 10.1042/BSR20220186 (PMC9254666; doi:10.1042/BSR20220186)

Supplementary Figure 1.

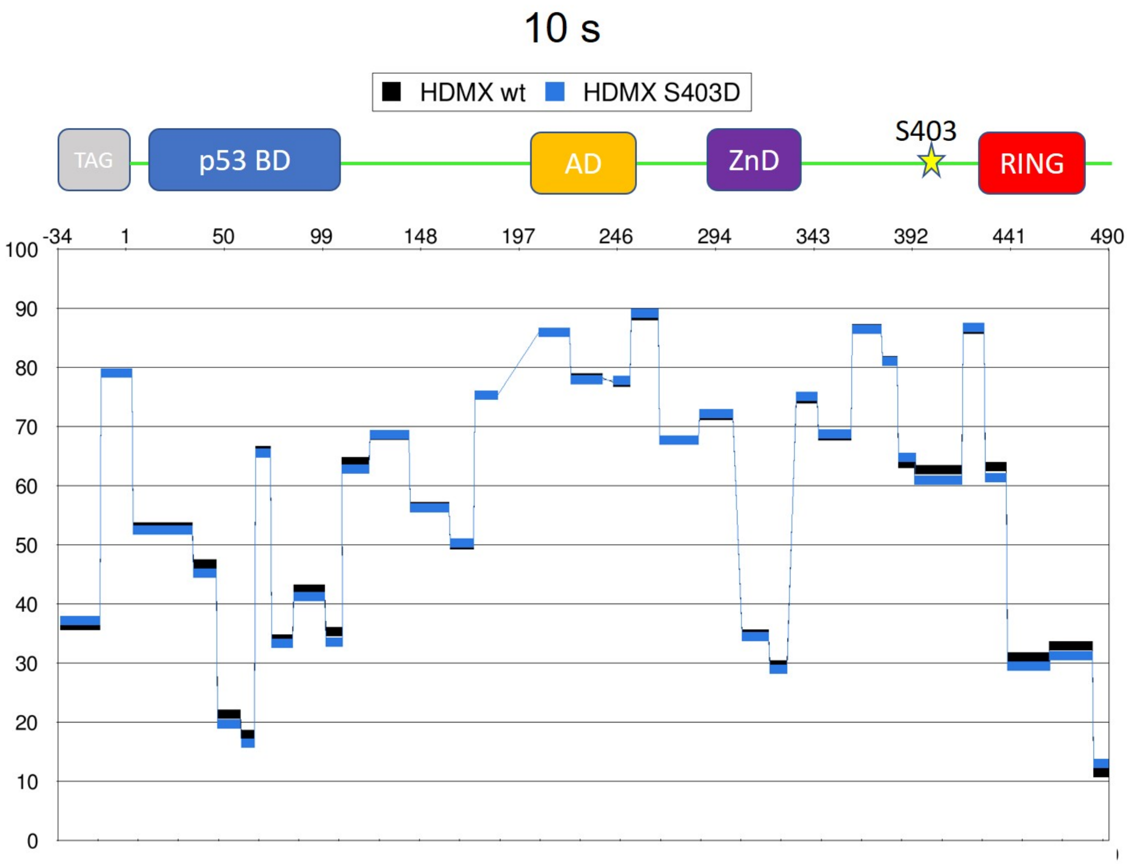

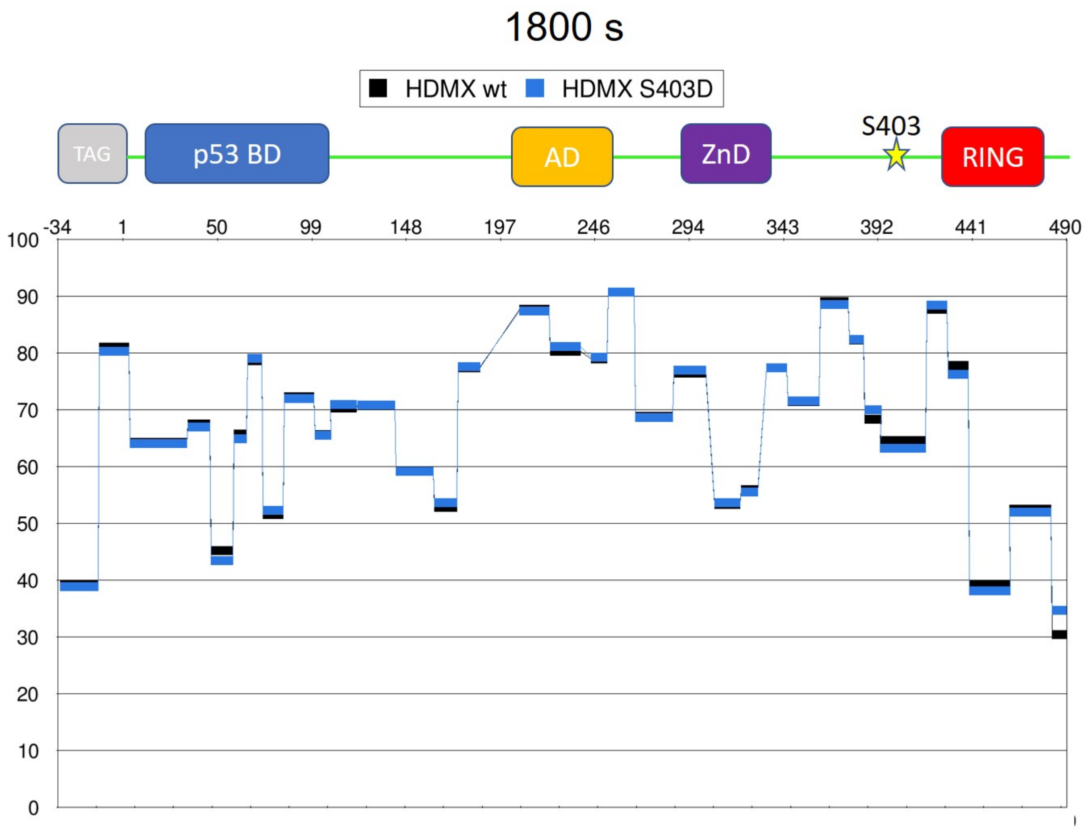

Supplementary Figure 1.

Effect of phosphomimetic mutation in Ser403 on the HDMX full-length structure. The upper panel shows HDX-MS of the full-length proteins HDMX (in black) and HDMX-S403D (in blue) after 10s (upper panel) and 1800s of (lower panel) deuteration. The data are plotted as % of deuteration of the peptide as a function of the numbering of the amino acids -34–490 after 10s or 1800s of incubation in the deuterated buffer. -34-1 amino acid residues corresponds to 6xHIS tag.

Supplementary Figure 2.

Figura 4A

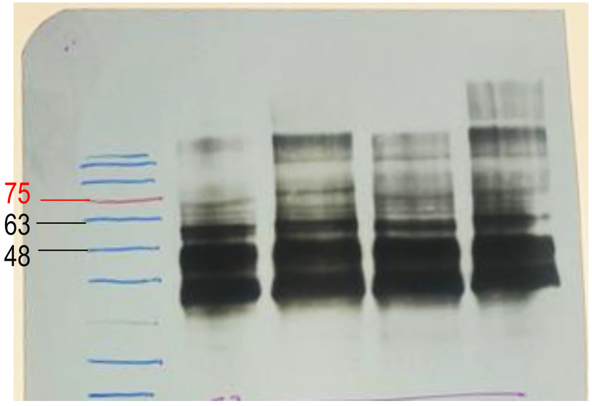

Anti p53 CM1

Figura 4B

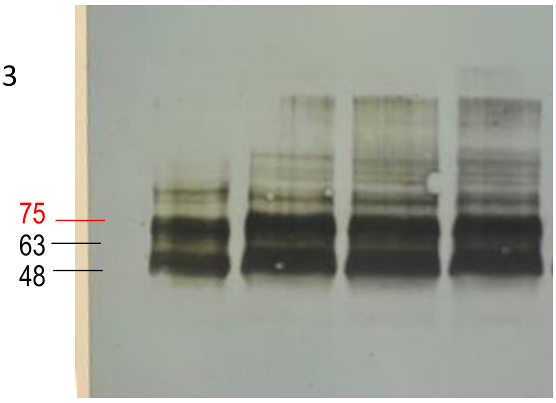

Figura 4C

p53	+	+	+	+	+	+	+
HDM2	-	+	-	-	-	-	-
HDMX	-	-	-	-	wt	S403D	-
p60	-	-	wt	S403D	-	-	-
(1-255)	-	-	-	-	-	-	+

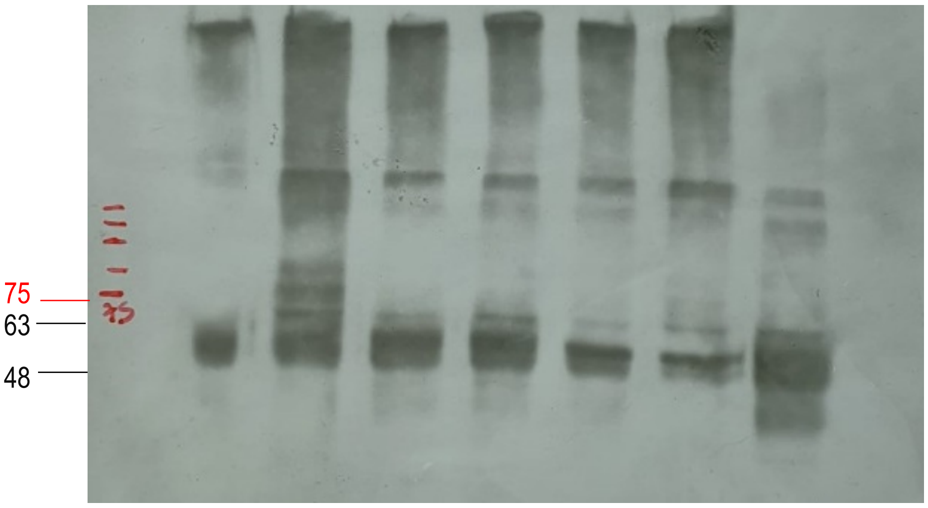

Anti p53 CM1

Figura 4 D

P53	+	+	+	+	+
HDMX	-			(322-490) wt	(322-490) S403D

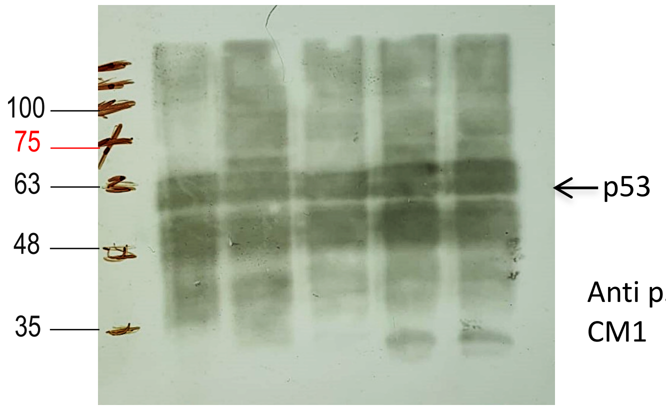

Anti p53 CM1

Supplementary Figure 2.

Figura 4 E

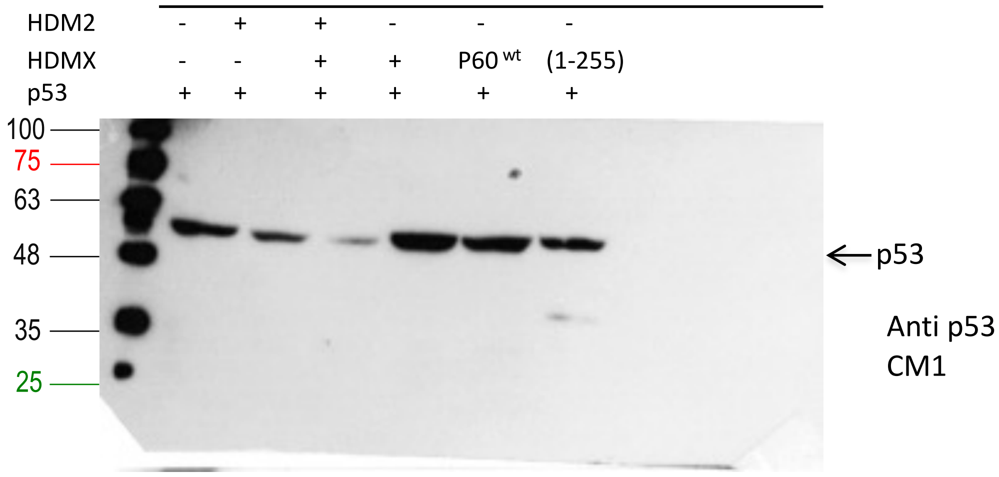

Figura 4 F

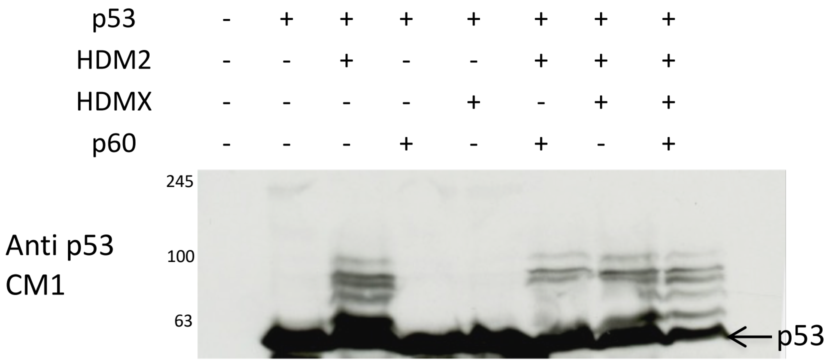

Supplement: Supplementary Figures S1-S2 [file BSR-2022-0186_supp.pdf]
